# Integrated Assessment of OCT, Multimodal Imaging, and Cytokine Markers for Predicting Treatment Responses in Retinal Vein Occlusion Associated Macular Edema: A Comparative Review of Anti-VEGF and Steroid Therapies

**DOI:** 10.3390/diagnostics14171983

**Published:** 2024-09-07

**Authors:** Marion R. Munk, Lala Ceklic, Richard Stillenmunkes, Varun Chaudhary, Nadia Waheed, Jay Chhablani, Marc D. de Smet, Anne Tillmann

**Affiliations:** 1 Augenarzt Praxisgemeinschaft Gutblick, 8808 Pfäffikon, Switzerlandanne-tillmann@hotmail.de (A.T.); 2 Department of Ophthalmology, Inselspital, Bern University Hospital, University of Bern, 3010 Bern, Switzerland; 3 Bern Photographic Reading Center, Inselspital, University Hospital Bern, 3010 Bern, Switzerland; 4 Department of Ophthalmology, Feinberg School of Medicine, Northwestern University, Chicago, IL 60208, USA; 5 Department of Surgery, McMaster University, Hamilton, ON L8S 4L8, Canada; 6 Department of Health Research Methods, Evidence, and Impact, McMaster University, Hamilton, ON L8S 4L8, Canada; 7 Department of Ophthalmology, Tufts University Medical School, Boston, MA 02111, USA; 8 Department of Ophthalmology, University of Pittsburgh School of Medicine Pittsburgh, Pittsburgh, PA 15213, USA; 9 MicroInvasive Ocular Surgery Center, 1005 Lausanne, Switzerland; 10 Department of Ophthalmology, Leiden University, 2311 EZ Leiden, The Netherlands; 11 New York Eye and Ear Infirmary of Mt Sinai, Icahn School of Medicine, New York, NY 10029, USA

**Keywords:** retinal vein occlusion, macular edema, central retinal vein occlusion, branch retinal vein occlusion, anti-VEGF therapy, corticosteroid treatment, imaging biomarkers, cytokine assessments

## Abstract

Retinal vein occlusion (RVO) is a significant cause of vision loss, characterized by the occlusion of retinal veins, leading to conditions such as central retinal vein occlusion (CRVO) and branch retinal vein occlusion (BRVO). Macular edema (ME), a prevalent consequence of RVO, is the primary cause of vision impairment in affected patients. Anti-VEGF agents have become the standard treatment, showing efficacy in improving visual acuity (VA) and reducing ME. However, a subset of patients exhibit a suboptimal response to anti-VEGF therapy, necessitating alternative treatments. Corticosteroids, which address inflammatory pathways implicated in ME, have shown promise, particularly in cases resistant to anti-VEGF. This review aims to identify biomarkers that predict treatment response to corticosteroids in RVO-associated ME, utilizing multimodal imaging and cytokine assessments. Baseline imaging, including SD-OCT and OCT-A, is essential for evaluating biomarkers like hyperreflective foci (HRF), serous retinal detachment (SRF), and central retinal thickness (CRT). Elevated cytokine levels, such as IL-6 and MCP-1, correlate with ME severity and poor anti-VEGF response. Early identification of these biomarkers can guide timely transitions to corticosteroid therapy, potentially enhancing treatment outcomes. The practical conclusion of this review is that integrating biomarker assessment into clinical practice enables personalized treatment decisions, allowing for earlier and more effective management of RVO-associated ME by transitioning patients to corticosteroid therapy when anti-VEGF agents are insufficient. Advanced diagnostics and machine learning may further refine personalized treatment strategies, improving the management of RVO-associated ME.

## 1. Introduction

Retinal vein occlusion (RVO) is a serious vision-threatening disease that occurs when a retinal vein is partly or completely occluded. In the case where the retinal vein is obstructed at or posterior to the optic nerve head, then it is a central retinal vein occlusion (CRVO). If there is an obstruction at a branch or a tributary of the central retinal vein, then we call it a branch retinal vein occlusion (BRVO). Hemi-retinal vein occlusion (HRVO) is an occlusion occurring at the level of the optic disc that commonly involves the venous drainage of half of the neurosensory retinal, thus either the superior or inferior hemifield. The global prevalence of an RVO in people aged 30–89 years is 0.77%. This implies that overall, 28 million people worldwide are affected and may suffer visual function decrease [1]. According to the literature, baseline visual acuity (VA) in RVO is significantly impaired, often below 20/40, which can substantially reduce vision-related quality of life [2]. Eyes with BRVO may show some improvement in VA without treatment, but achieving clinically significant improvement beyond 20/40 is rare. In cases of CRVO, poor baseline VA (less than 20/40) tends to deteriorate further over time, as time leads to a more ischemic state within the retinal vasculature [3,4]. While several factors can lead to vision loss due to RVO, macular edema (ME) secondary to RVO (RVO-ME) is the most prevalent cause [5,6].

ME occurs in 5–15% of BRVO cases within one year of the initial onset, and spontaneous resolution is achieved in less than half of the cases [4]. No differences in the frequency of ME occurrence between superior and inferior BRVO have been reported so far. No differences in VA at initial presentation between inferior and superior involvement have been found for either temporal major BRVO or macular BRVO [7]. Spontaneous improvement in visual acuity after 3 years, with visual acuity reaching 20/40 or better, was observed in 34% of cases. The recovery of VA is attributed to the resolution of edema mainly due to the development of collateral vessels that aid in venous drainage [8].

Compared to patients with BRVO, those with CRVO-associated ME typically experience more severe vision loss, which often worsens over time despite treatment. Patients with non-ischemic CRVO usually have a relatively mild disease course, with ME resolving in about 30% of cases and pathological neovascularization occurring infrequently. Conversely, patients with ischemic CRVO rarely showing visual improvement and have a high risk of pathological neovascularization, with neovascular glaucoma developing in approximately 25% of these cases within a few months of onset [3].

The exact pathogenesis of ME in RVO remains unclear. However, contributing factors include obstructed veins leading to increased intraluminal pressure, which results in reduced venous blood flow velocity, varying degrees of retinal capillary nonperfusion, and retinal hypoxia [9]. Another significant contributing factor is inflammation [10].

Since long-term untreated RVO-ME results in the irreversible deterioration of visual function, it is crucial to detect and treat ME promptly [4].

Table 1 provides an overview of various therapeutic strategies for patients with macular edema due to retinal vein occlusion, summarizing relevant study data. The Branch Vein Occlusion Study (BVOS) demonstrated that focal laser photocoagulation significantly improves VA in patients with ME resulting from BRVO; in patients with VA ≤ 20/40, 65% of treated eyes gained two or more lines from baseline, compared with 17% of non-treated eyes [8]. In cases of CRVO, laser photocoagulation did not lead to enhanced vision [11]. Before the advent of anti-VEGF therapy, focal laser photocoagulation was the primary treatment for ME due to BRVO. Comparisons between intravitreal anti-VEGF therapy and focal laser photocoagulation revealed that anti-VEGF therapy produced significantly better outcomes [12]. The introduction of anti-VEGF agents has since demonstrated rapid vision improvement and ME reduction, becoming the standard of care in both BRVO-ME and CRVO-ME. Currently, three anti-VEGF agents (ranibizumab, aflibercept, and faricimab) have been approved for the treatment of RVO-associated ME by the FDA (Food and Drug Administration) and two (ranibizumab and aflibercept) by the EMA (European Medicine Agency). Another VEGF inhibitor (bevacizumab) is often used “off-label” in clinical practice [13].

Multicenter, randomized pivotal trials have demonstrated the efficacy and safety of intravitreal ranibizumab at 0.5 mg (BRAVO), aflibercept at 2.0 mg (VIBRANT), and faricimab at 6 mg (BALATON) in patients with macular edema (ME) secondary to BRVO [14,15]. The BRAVO study demonstrated a significant visual gain at 6 months after 5.7 injections of ranibizumab and at 12 months after 8.4 injections of ranibizumab [16]. In the VIBRANT study, the treatment of ME induced by BRVO was optimized with 5.7 injections of aflibercept after 6 months and 9 injections after 12 months [12]. In both studies, visual gain after 12 months ranged from 18.3 to 17.1 letters. The Balaton study reported a 16.9- and 17.5-BCVA-letter gain in the faricimab and aflibercept groups at 24 weeks with 4 weekly injections [17].

For patients with ME secondary to CRVO, the efficacy and safety of intravitreal ranibizumab at 0.5 mg (CRUISE), aflibercept at 2.0 mg (COPERNICUS/GALILEO), and faricimab at 6 mg (COMINO) have also been demonstrated [16,17,18,19]. The CRUISE study showed a 14.9-letter gain for the ranibizumab group compared with 0.8 letters in the sham group after 6 months [16]. In the COPERNICUS study, a 15-letter gain was found in 56% of aflibercept-treated eyes compared to 12% of eyes in the sham group after 6 months [18]. Similarly, in the GALILEO study, both a 15-letter gain in VA compared to baseline and central foveal thickness reduction were significantly greater in the treated group versus the sham group [19]. The BCVA gains from the baseline to week 24 with faricimab were noninferior versus aflibercept in COMINO (+16.9 letters vs. +17.3 letters) with a comparable safety profile [17].

However, some patients experience recurrent or persistent ME and require repeated anti-VEGF injections [20,21]. Menke et al. defined low responders to anti-VEGF treatment as cases in which VA does not improve by more than 6 letters from baseline or central retinal thickness (CRT) does not decrease by more than 30% from baseline. They found that approximately 30% of patients with ME due to RVO demonstrated a low response to ranibizumab after one year of treatment [22]. During the 4-year follow-up of the RETAIN study, approximately 50% of patients continued to have unresolved ME [21]. These numbers highlight that the ME and underlying mechanisms might not be satisfactorily controlled by the exclusive suppression of VEGF.

In acute RVO, the ischemia caused by vascular obstruction leads to an increase in the expression and secretion of VEGF. Further inflammatory factors play an important role in RVO and affect the progression and outcome; soluble cytokines and other permeability factors such as interleukin (IL) 6 and 8, monocyte chemoattractant protein-1, and platelet-derived growth factor (PDGF) are significantly elevated to variable degrees. Both VEGF and IL-6 levels are found to be correlated with the severity of ME and extent of retinal ischemia (capillary nonperfusion) [23,24,25,26,27]. Even if the primary venous obstruction is resolved, such as through collateral formation, ME can persist for an extended period due to a self-perpetuating cycle. This cycle involves VEGF-induced vascular permeability, resulting in macular edema, capillary damage, and retinal ischemia, which in turn stimulate the further release of VEGF and other inflammatory cytokines, leading to chronic macula edema [28,29]. In chronic, recurrent, and persisting RVO-associated ME, inflammatory proteins and cytokines (such as MCP-1 (chemokine monocyte chemoattractant protein), ICAM-1 (intercellular adhesion molecule 1), interleukin 6, and interleukin 8 (IL-6 and IL 8) play an important role apart from VEGF and PDGF (platelet-derived growth factor) [24]. Corticosteroids not only inhibit the expression of inflammatory cytokine and adhesion molecules but also that of VEGF [30]. Their anti-inflammatory effects show promise in RVO, even in cases resistant to anti-VEGF treatment and in the chronic phase of RVO-associated ME. The hypoxic conditions caused by vascular stasis and capillary closure might also result in structural damage and the loss of Mueller cells. These cells are crucial for maintaining the functional and structural homeostasis of the human retina, particularly in regulating the intra- and extracellular fluid balance and the structural integrity of the foveal region [31,32]. Studies using mouse models of BRVO have underscored the pivotal role that Mueller cells play in managing the reabsorption of intraretinal fluid [33]. When Mueller cells are compromised, this can lead to an increase in permeability and pro-edema factors, disrupting the homeostasis of intraretinal fluid [34,35].

Intravitreal steroid treatment for ME secondary to RVO is generally used as a second-line therapy [36]. The efficacy and safety of corticosteroids in the treatment of RVO have been demonstrated in two major clinical trials. The SCORE study compared 1 mg and 4 mg doses of preservative-free intravitreal triamcinolone (IVTA) to standard care (observation for CRVO and grid laser for BRVO). After one year, 27% of those receiving 1 mg IVTA and 26% of those with 4 mg IVTA showed VA improvement of three or more lines versus 7% in the observation (CRVO) group, with improvements persisting into the second year. However, higher cataract formation and IOP were noted in the steroid groups [37]. The GENEVA trial assessed a dexamethasone intravitreal implant (Ozurdex) in 1267 patients with ME from BRVO or CRVO. Both the 0.7 mg and 0.35 mg implants led to significant VA improvements compared to the sham group (*p* < 0.001). Elevated IOP was observed in 16% of patients at day 60 but normalized by day 180, with no significant difference in cataract formation or surgery compared to the sham group [38] during the 6-month duration of the study.

Another pivotal, randomized, sham-controlled, multicenter study from China confirmed the safety and efficacy of a dexamethasone intravitreal implant (0.7 mg) for the treatment of ME secondary to RVO in this population. At month 2 (peak effect), the percentage of patients with a ≥15-letter BCVA improvement from baseline was significantly higher in the dexamethasone group (35%) compared to the sham group (12%) (*p* < 0.001). The most frequently observed adverse event following treatment was an increase in IOP. These IOP elevations were typically managed with topical medications. By the fourth month, the average IOP had returned to normal levels, and no patients needed incisional glaucoma surgery [39].

**Table 1:** Therapeutic strategies for retinal vein occlusion associated macular edema.

Treatment	Studies	Methods	Results
Laser Therapy:			
Focal laser photocoagulation	BVOS (1984) [8]	BRVO ME patients with VA ≤ 20/40: grid pattern laser vs. observation	- VA gain in ≥ 2 lines in 65% vs. 37% of untreated - no VA gain for CRVO (CVOS study)
Anti-VEGF agents:			
- Ranibizumab	BRAVO [16] (BRVO) CRUISE [16] (CRVO)	0.3 mg vs. 0.5 mg vs. sham 0.3 mg vs. 0.5 mg vs. sham	at 6 months: +16.6 vs. +18.3 vs. +7.3 significant letter gain at 6 months: +12.7 vs. +14.9 vs. +0.8 significant letter gain
- Aflibercept	VIBRANT [12] (BRVO) COPERNICUS [18] (CRVO) GALILEO [19] (CRVO)	2.0 mg vs. grid laser 2.0 mg vs. sham 2.0 mg vs. sham	at week 24: +17.0 vs. +6.9 significant letter gain at week 24: +17.3 vs. −4.0 significant letter gain at week 52: +16.9 vs. +3.8 significant letter gain
- Faricimab	BALATON [17] (BRVO) COMINO [17] (CRVO)	6.0 mg vs. 2.0 mg aflibercept 6.0 mg vs. 2.0 mg aflibercept	at week 24: +16.9 vs. +17.5 noninferior letter change at week 24: +16.9 vs. +17.3 noninferior letter change
- Bevacizumab		Off-label use	
Steroids			
- TA	SCORE [37]	1 mg vs. 4 mg vs. standard of care (grid laser/observation)	at 12 months: for BRVO (grid laser) +5.7 vs. +4.0 vs. +4.2 no significant letter gain for CRVO (observation) −1.2 vs. −1.2 vs. −12.1 signifcant letter change higher cataract formation and IOP in TA groups (4 mg > 1 mg)
- DEX	GENEVA [38]	0.7 mg vs. 0.35 mg vs. sham	- time to ≥15-letter gain shorter in DEX implant groups vs. sham (*p* < 0.001) - no significant difference in cataract incidence or IOP increase by day 180

Abbreviations: BVOS: branch vein occlusion study; BRVO: branch retinal vein occlusion; ME: macular edema; VA: visual acuity; CRVO: central retinal vein occlusion; CVOS: central vein occlusion study; TA: triamcinolone; DEX: dexamethasone; IOP: intraocular pressure.

Unfortunately, there have been only a few head-to-head studies comparing the efficacy of anti-VEGF treatments versus corticosteroids for recurrent ME secondary to RVO.

The COMRADE-B and COMRADE-C studies were two 6-month, head-to-head, phase IIIb, multicenter, randomized, double-masked trials designed to compare the efficacy and safety of 0.5 g ranibizumab on a PRN basis vs. a single injection of a 0.7 mg dexamethasone intravitreal implant for macular edema secondary to retinal vein occlusions. COMRADE-B focused on BRVO and COMRADE-C on CRVO. Both medications effectively reduced macular thickness and initially improved BCVA, with notable enhancements observed at months 1 and 2, showing no discernible differences between the two drugs. However, by month 3, there was a significant difference in mean BCVA improvement from baseline, favoring ranibizumab over dexamethasone (+16.2 vs. +9.3 letters in COMRADE-B and +16.0 vs. +7.0 letters in COMRADE-C). This trend continued at month 6, with ranibizumab demonstrating a greater mean change in BCVA compared to dexamethasone (+17.3 vs. +9.2 letters in COMRADE-B and +16.9 vs. –0.7 letters in COMRADE-C). These findings underscore the superiority of the ranibizumab regimen administered as needed, guided by individualized stabilization criteria, over a single injection of Ozurdex^®^ throughout a 6-month period [40,41]. A real-life study (42 treatment-naïve patients with CRVO) exhibited a similar pattern of outcomes as those observed in the clinical trials. However, it yielded inferior results in terms of improvement in VA [42]. Another small study with 64 patients showed no disparity in terms of BCVA or reduction in CRT for ranibizumab (*n* = 32) vs. Ozurdex patients (*n* = 32). However, there was a statistically significant elevation in IOP among the patients treated with Ozurdex [43]. The OMAR study was the first designed to analyze the efficacy and cost-effectiveness of a dexamethasone intravitreal implant and intravitreal TA injection for the treatment of recalcitrant ME in patients with RVO [44]. The research comprised 38 individuals with resistant ME stemming from BRVO and 36 individuals with refractory ME associated with CRVO. Patients received an average of 5.16 ± 1.85 (but at least 3) bevacizumab injections prior to starting intravitreal steroid treatment. Subsequently, the treatment approach was shifted to an intravitreal steroid, either triamcinolone acetonide at a dosage of 4 mg/0.1 cc or a 0.7 mg dexamethasone implant, due to inadequate response to treatment and persistent ME. Switching to steroids improved central macular thickness significantly. While both steroid treatments led to a notably improved anatomic outcome after their initiation in both CRVO and BRVO, there was no considerable change in the mean functional outcome (BCVA). This observation might be attributed to the diminished visual potential resulting from prolonged chronic ME, ischemia, and concomitant irreversible damage to photoreceptors. There was no difference between triamcinolone and dexamethasone with respect to anatomic or functional outcomes. The percentage of cases requiring IOP-lowering medications were consistent with the findings from the SCORE and GENEVA studies. No statistically significant difference was observed between the triamcinolone and dexamethasone groups regarding increases in IOP. The average period between anti-VEGF injections before the commencement of intravitreal steroid injection was 1.5 months. Following the introduction of steroid treatment, the average interval between injections extended to over 4 months. The authors suggested that commencing steroid treatment earlier in the ME treatment process might have led to enhanced functional outcomes. Determining the appropriate juncture to classify a patient as unresponsive to anti-VEGF treatment and deciding when to transition from anti-VEGF to steroid therapies are questions that require further clarification.

Substantial evidence indicates a significant involvement of inflammatory mediators in the pathophysiology of RVO, which supports the use of corticosteroids in cases with a clear inflammation-driven mechanism and also in cases with chronic resistant ME. Corticosteroids act through diverse mechanisms: reducing the synthesis of inflammatory mediators, permeability, and adhesion molecules, coupled with a decrease in VEGF levels [30]. Their use in RVO targets more pathways than only anti-VEGF, which specifically targets a segment of the angiogenetic cascade. Additionally, in a recent study, steroids lead to a higher elevation of arteriovenous oxygen levels in the retina as compared to anti-VEGF. This suggests enhanced retinal oxygenation following the injection of a dexamethasone implant [45].

The aim of this review article is to investigate which baseline multimodal imaging biomarkers and intraocular cytokines in patients with macular edema secondary to RVO can predict a positive response to the early addition of, or switch to, steroid therapy. Through an integrated assessment of OCT, multimodal imaging, and cytokine markers, this paper seeks to provide guidance on when a switch to corticosteroids may be beneficial and when an early transition from the anti-VEGF gold-standard treatment to intravitreal steroids may offer advantages in optimizing treatment outcomes for patients with RVO.

## 2. Method

A comprehensive literature search was conducted on PubMed and Google Scholar for English-language publications using the keywords “Retinal vein occlusion” combined with “Treatment”, “Diagnosis”, “Biomarkers”, “Management”, and “Outcomes”. Additional pertinent studies were identified by examining references cited in the selected articles. We also reviewed published national and international guidelines, case reports, case series, meta-analyses, and retrospective studies. The references included in this article were evaluated for relevance through a panel discussion and consensus. An initial draft was prepared and circulated among all authors, who provided feedback, suggestions, and comments. After incorporating the panel’s input, the manuscript was revised and finalized. Consensus on the content was reached through meticulous review and agreement among all authors. Any discrepancies were resolved through co-author discussions, supported by detailed reference searches to substantiate evidence-based statements.

## 3. Results

### 3.1. Inflammation as a Crucial Pathomechanism of ME in RVO

ME is the main reason for low vision in patients with RVOs, and one main factor contributing to this condition is hypoxia. However, ME can also occur in mild non-ischemic BRVO, where hypoxia may not be the primary cause. Traditionally, increased levels of VEGF have been correlated with ischemic areas. ME, which is driven by VEGF, responds very well to anti-VEGF agents; however, approximately 30% of patients with ME due to RVO exhibit a poor response to anti-VEGF treatment after the loading dose and after one year of treatment [22,24]. The main driver of ME due to RVO that is primarily not driven by VEGF is inflammation. In cases of RVO, the obstruction of the retinal vein leads to an increase in venous pressure, resulting in retinal hemorrhages, vascular leakage, and ischemia [10]. While VEGF plays a significant role in mediating vascular permeability and neovascularization, contributing to ME, inflammation is another critical pathway that contributes to the pathophysiology of ME in RVO. Structural damage and loss of Mueller cells could also play an important role here. Growing evidence shows that Mueller glia play critical roles in the regulation of retinal inflammation. They express various receptors for cytokines and release cytokines to regulate inflammation [46]. Patients with RVO have higher values of aqueous flare, an index of inflammation, which correlates with higher levels of intraocular cytokines [24,47]. Intraocular levels of growth factors, chemokines, and adhesion molecules are also increased in RVO. Inflammation appears to play a crucial role in the pathogenesis of ME in RVO. Analyses of inflammatory cytokines (e.g., IL-6, IL-8, ICAM-1, and MCP-1) and their correlation with imaging biomarkers should help us identify cases with more inflammation-driven ME [22,24,48,49,50,51]. Given that cytokine profiling and aqueous humor sampling are not commonly performed in routine clinical practice, it becomes essential to depend on imaging biomarkers that correlate with inflammation and ischemia. These biomarkers can provide valuable insights for selecting the most appropriate treatment strategy.

### 3.2. Cytokines Involved in RVO and RVO-Associated ME

An intricate interplay of cytokines and chemokines is observed in the course of RVO. These signaling molecules, produced by retinal cells (particularly microglia and Mueller cells [46]), immune cells, and endothelial cells, play pivotal roles in orchestrating inflammation, angiogenesis, and tissue remodeling within the retinal microenvironment. Unraveling the network of cytokines and chemokines associated with RVO will lead to a deeper understanding of the disease mechanisms and ultimately to better therapy. Similar cytokine profiles are found in both CRVO and BRVO. The concentrations of respective cytokines (e.g., IL-8, ICAM-1, Ang 2, and MCP-1) tend to be higher in CRVO compared to BRVO [52] and are also generally higher under ischemic vs non ischemic conditions [53].

#### 3.2.1. Interleukin (IL)-6

Interleukin (IL)-6 is a soluble mediator derived from macrophages, activated T lymphocytes with multiple functions in inflammation, immune response, and hematopoiesis. It is synthesized locally by Mueller and vascular endothelial cells in the initial stages of inflammation. IL-6 promotes the specific differentiation of naïve CD4+T cells and, through its interaction with T cells, is involved in autoimmune and chronic inflammatory diseases [54]. Apart from playing a crucial role in the initiation of uveitis and consequent ME via these inflammatory pathways, IL-6 can also contribute to the occurrence of ME through alternative mechanisms. IL-6 stimulates the generation of VEGF and promotes vascular leakage by reducing the expression of tight junction proteins in retinal endothelial cells. Patients with CRVO and ischemia have significantly higher vitreous fluid levels of IL-6 and VEGF, and these are significantly correlated with the extent and severity of ischemia and ME [24,49]. IL-6 also plays a protective role for retinal ganglion cells [55]. IL-6 mRNA expression is increased in hypoxia-exposed cultured endothelial cells [56]. Increased IL-6 levels are significantly associated with inner central retinal thickness (CRT), nonperfused areas, serous retinal detachment, and aqueous flare in RVO patients [26,57,58]. Higher levels of IL-6 were indicative of slower recurrence of ME after anti-VEGF treatment [59].

#### 3.2.2. Interleukin (IL)-8

Interleukin 8 (IL-8) is a potent chemoattractant that activates neutrophils and T cells. It is highly expressed in vascular endothelial cells exposed to hypoxia and oxidative stress [24,60,61] and retinal pigment epithelial cells (HRPEs). It is elevated in aqueous humor, and in vitro, both in CRVO and BRVO [48]. In CRVO, IL-8 is expressed in the retina and plays an important role in activating other proinflammatory cytokines and increasing leukocyte adhesion to the vascular endothelium [62]. VEGF promotes its secretion from endothelial cells [63]. Intraocular levels of IL-8 decrease with resolution of ME in response to various treatments (anti-VEGF [48,64,65,66], corticosteroids [52], and vitrectomy [67]). There is speculation that levels of IL-8 could predict the recurrence of ME following intravitreal treatment [26]. ME severity was notably higher with elevated baseline levels of IL-8. Additionally, CRT before treatment showed a positive correlation with IL-8 levels in the aqueous humor [59]. IL-8 levels also had a strong correlation with baseline aqueous flare, nonperfused areas, and VA [26,57]. Another study indicated that the number of hyperreflective foci (HRF) in persistent ME significantly decreased following anti-VEGF treatments, and a positive correlation was found between IL-8 levels and the number of HRF observed via SD-OCT [68].

#### 3.2.3. Interleukin-12 (IL-12) and Interleukin-13 (IL-13)

IL-12 and IL-13 are key pro-inflammatory cytokines involved in pathogenesis of allergy, cancer, and tissue fibrosis, and they are both elevated in RVO [26]. There appears to be a negative correlation between intraocular IL-12/IL-13 with retinal ischemia, CRT, and serous retinal detachment [69,70]. Some studies have reported a significant association between aqueous levels of IL-12 and a refractory response to anti-VEGF in BRVO. Higher levels of IL-13 were also associated with an insufficient response to anti-VEGF [57,65], hinting at a more inflammatory-driven pathogenesis in these cases. A significant association between intraocular levels of IL12 and IL 13 and the aqueous flare value could not be found [58,71].

### 3.3. Growth Factors

#### 3.3.1. Vascular Endothelial Growth Factor (VEGF)

VEGF is a key factor in angiogenesis and increased vascular permeability. In RVO, it is the main cytokine in response to ischemia and is responsible for neovascularization [72,73]. Due to hypoxia or blood stasis, there is an increase in vascular permeability, leading to the exudation of VEGF. VEGF induces ICAM-1, leading to leukocyte stasis and alterations in vascular permeability [74]. In the context of ME secondary to BRVO, VEGF together with ICAM-1, IL-6, and MCP-1 disrupt the blood–retinal barrier [75]. In neovascular glaucoma due to RVO, the VEGF-VEGFR2 pathway suppresses occludin, causing damage to intercellular tight junctions, and activates MMP-9, resulting in the destruction of the blood–retinal barrier [76]. Moreover, VEGF leads to exudation, promoting adhesion hyperplasia and angiogenesis, ultimately resulting in neovascular glaucoma and high intraocular pressure [77,78]. VEGF levels are significantly increased in patients with RVO-associated ME and were positively correlated with ME and serous retinal detachment [26,79]. Higher VEGF levels prior to treatment with anti-VEGF led to a faster decrease in CRT [59]. In a study involving patients with ME secondary to BRVO and treated with ranibizumab, it was observed that aqueous humor levels of VEGF significantly decreased over time, even though ME recurred following anti-VEGF therapy [64]. No notable changes in other cytokines such as IL-6, IL-12, and IL-13 were found. By contrast, following the use of a dexamethasone implant, no significant changes in VEGF aqueous concentrations in BRVO and CRVO were observed, while proinflammatory markers and chemokines such as MCP-1, IL17-E, and IL-1alpha significantly decreased during the treatment period of 6 months [52]. These results suggest that persistent inflammation might be a more important contributor as compared to anti-VEGF to the recurrence of ME in BRVO patients after anti-VEGF therapy and that incorporating a steroid into the anti-VEGF therapy regimen could be a more effective approach to prevent recurrence [64]. A correlation exists between the VEGF concentration in ME secondary to BRVO and the size of nonperfusion detected on fluorescein angiography [80]. VEGF levels were also associated with increased macular and peripheral ischemia as well as increased macular and peripheral leakage on fluorescein angiography [81].

#### 3.3.2. Platelet-Derived Growth Factor (PDGF)

The PDGF family comprises four ligands: PDGF-A, PDGF-B, PDGF-C, and PDGF-D. These ligands can function as homodimers or, in the case of PDGF-AB, as a heterodimer. PDGF-AA, -AB, -BB, and -CC specifically activate the PDGF receptor-α (PDGFRα), while PDGF-BB and -DD are known to bind to PDGFRβ [82]. Within the normal eye, PDGF-A is produced by both neurons and astrocytes [83]. Together with PDGFRα, it plays a crucial role in attracting astrocyte precursors to the retina and guiding their development [84]. This interaction between PDGF-A and PDGFRα is essential for regulating the quantity and distribution of astrocytes in the retinal tissue. The maintenance of retinal blood vessels relies on PDGF-B signaling through PDGFRβ. Pericytes, which express PDGFRβ, attach to blood vessels through PDGF released by endothelial cells [82,85,86]. Despite its protective function, PDGF-A collaborates with VEGF to stimulate neovascularization, presenting itself as a target for angiogenesis in conjunction with VEGF [87]. In RVO, there is a significantly increased concentration of PDGF-AA in aqueous humor, and it contributes to inflammation [62,88]. Intraocular PDGF-AA levels were reported to be higher for CRVO compared to BRVO [53] and positively correlated to retinal ischemia, ME, and subfoveal serous retinal thickness [26,69,70].

#### 3.3.3. Monocyte Chemoattractant Protein (MCP-1)

MCP-1 promotes monocyte chemotaxis and the phosphorylation of tight junction proteins. The MCP-1 level correlates with morphologic changes, such as the height of serous retinal detachments, higher CRTs, increased macular ischemia, and the size of nonperfused areas in RVO [26,69,70,81]. Its levels are particularly elevated in CRVO prior to any treatment [72]. Retinal ischemia, atherosclerosis, and oxidative stress increase the expression of MCP-1 [89]. MCP-1 seems to be involved in microvascular endothelial injury, leading to the breakdown of the inner blood–retinal barrier. VEGF ramps up the expression of MCP-1, while MCP-1, by recruiting eosinophils, leads to further increases in VEGF production [26,90]. Anti-VEGFs (bevacizumab and ranibizumab) have no significant effect on MCP-1 levels despite a lessening of edema and improvements in vision [27,91]. However, the use of intravitreal dexamethasone implants leads to a reduction in MCP-1 in proportion to improvements in ME in both BRVO and CRVO. A rise in MCP-1 preceded the recurrence of ME and could therefore potentially guide retreatment [52].

#### 3.3.4. Intercellular Adhesion Molecule 1 (ICAM-1)

ICAM-1 increases in response to retinal ischemia, promoting leukocyte rolling and adhesion to vessel walls, resulting in leukostasis and blood stagnation [13,15,89]. This process contributes to microvascular endothelial injury and the breakdown of the inner blood–retinal barrier [89]. ICAM-1 is significantly elevated in both BRVO and CRVO and positively correlates with CRT, the height of serous retinal detachment, and the degree of retinal ischemia [26,69]. ICAM-1 is not significantly suppressed by intravitreal anti-VEGFs [27,58]. A significant decrease in aqueous ICAM-1 levels is observed after intravitreal triamcinolone. Like MCP-1, ICAM-1 levels may have a predictive role for ME recurrence [49,71].

#### 3.3.5. Interferon-Inducible 10-kDa Protein (IP-10)

IP-10 inhibits the proliferation of endothelial cells and induces their apoptosis [24,92]. IP-10 is increased in vitreous fluid in RVO and inhibits VEGF-induced endothelial motility [93]. It is secreted by macrophages, endothelial cells, and fibroblasts. IP-10, by attracting macrophages, dendritic cells, and T cells, contributes to T-helper type 1 immune responses and activates cell-mediated immunity in cases of CRVO [24]. The IP-10 concentration decreases during dexamethasone treatment and is associated with CRT reduction [52].

#### 3.3.6. Pentraxin 3 (PTX 3)

PTX 3 is an acute-phase protein belonging to the family of acute inflammatory response proteins such as C-reactive protein (CRP) and serum amyloid P. PTX 3 is produced by the retinal pigment epithelium in response to proinflammatory cytokines and mediates the retinal inflammatory response. PTX3 levels are significantly higher in patients with BRVO and CRVO [24,94,95] and were found to be reduced in response to a dexamethasone implant [30].

#### 3.3.7. Erythropoetin (EPO)

EPO, in addition to its hematopoietic effects, exhibits neuroprotective properties in the retina [21,89]. Ocular EPO levels are elevated in patients with CRVO, especially in the ischemic subtype, compared to normal eyes [24,26,96,97]. EPO is upregulated in response to retinal ischemia, where it exerts neuroprotective effects against ischemia–reperfusion injury and light-induced retinal degeneration [24,96]. There is also evidence that it will favor the dilatation of retinal vasculature, possibly promoting better oxygenation [98]. Higher vitreal EPO levels correlate with increased vitreal levels of VEGF and more severe macular edema [24,97,99].

#### 3.3.8. Angiopoietin-2 (ANG2)

ANG2 is a proangiogenic cytokine that interacts with the Tie2 receptor on endothelial cells in blood vessels. As an important contributor to the angiogenesis pathway, ANG2 acts as a vessel-destabilizing agent by competing with Ang1 and inhibiting Tie2. Similar to VEGF, ANG2 is upregulated in response to hypoxia, and its ocular levels are significantly elevated in eyes affected by retinal vascular diseases such as RVO, wet AMD, and diabetic retinopathy [100,101,102].

The breakdown of the endothelial barrier is a critical event in retinal eye diseases, with edema being a major contributor to retinal pathology. ANG2 appears to exacerbate VEGF-A-induced barrier breakdown, further driving the disease process [100]. In RVO patients, aqueous levels of ANG2 were found to be significantly higher than their controls and correlated with the central retinal thickness (CRT) [103].

### 3.4. Imaging Biomarkers in RVO-Associated ME

In the following section, we will focus on predictive biomarkers that are employed to predict the effect and impact of a particular treatment. While prognostic biomarkers can be used to forecast future functional outcome irrespective of the treatment, predictive biomarkers are beneficial in orienting the optimal choice of treatment for the patient. Predictive biomarkers often also have a prognostic value. Table 2 provides an overview of important predictive biomarkers regarding treatment response to anti-VEGF or steroids in patients with ME due to RVO. Table 3 summarizes prognostic biomarkers for poor visual outcomes in patients with ME due to RVO.

Based on the current treatment options and our previous discussion of cytokine profiles in RVO, patients with high VEGF will benefit most from anti-VEGF treatment, while patients with high inflammatory cytokines will likely benefit more from intravitreal steroid treatment. As cytokine profiling and aqueous humor taps are usually not performed in busy clinical settings, we need to rely on predictive biomarkers that correlate to disease states, inflammatory status, and ischemia to help us chose the appropriate treatment.

#### 3.4.1. Cardinal Features in Spectral Domain (SD) Optical Coherence Tomography (OCT)

OCT is the most commonly used imaging modality in RVO-associated CME. It allows for, in a noninvasive way, the identification of many morphological changes. It is used as a diagnostic tool and to observe individual treatment responses.

**Central retinal thickness (CRT)** is a critical biomarker in assessing the severity and progression of retinal vascular disorders. It is quantitatively assessed as the average thickness of the retina from its inner- to outer-most boundary within the central 1 mm zone, as determined across multiple scans [36]. Clinically, an increase in CRT is directly linked to vision impairment during the acute phase of RVO-related ME [20,36,130,131]. However, this association diminishes over time. A decrease in CRT does not necessarily correlate with better visual outcomes [132,133,134,135]. This disconnect highlights the multifactorial nature of vision restoration, where factors beyond retinal thickness, such as photoreceptor integrity and neuroretinal remodeling, play crucial roles. As mentioned earlier, CRT positively correlates with the vitreous fluid levels of VEGF and several inflammatory cytokines such as IL-6, IL-8, MCP-1, ICAM-1, IP-10, and ANG2 [52,69,103]. Higher VEGF levels prior to treatment are indicative of a faster decrease in the CRT under anti-VEGF treatment, suggesting that VEGF-driven mechanisms are key drivers of ME in these cases [59].

In cases of CRVO and BRVO that were unresponsive to at least five anti-VEGF injections, dexamethasone implants have demonstrated both efficacy and safety. The treatment resulted in a significant CRT reduction, decreasing from a baseline of 482.92 ± 139.99 μm to 295.82 ± 135.48 μm in the BRVO group and from 669.70 ± 203.20 μm to 549.90 ± 191.26 μm in the CRVO group 12 months after the initial dexamethasone implant. Each patient received an initial intraocular dexamethasone implant, with the procedure being repeated at a 6-month interval “as needed” [104]. These findings suggest that in cases where anti-VEGF therapy fails, the switch to intravitreal steroids can effectively reduce CRT and potentially improve visual outcomes.

Furthermore, patients who switched from anti-VEGF to intravitreal steroids experienced a mean letter gain of four letters and an additional central foveal thickness decrease of 170 micrometers. In contrast, switching from steroids to anti-VEGF in patients with persistent ME due to RVO had no effect on CRT or visual acuity [105]. This observation reinforces the notion that in certain patients, ME may be driven by non-VEGF mechanisms, and that such patients may benefit more from steroid therapy than from additional anti-VEGF treatments.

Interestingly, an initial CRT of >570 micrometers was found to be associated with recurrence of ME under intravitreal bevacizumab injection [106]. Thicker pre-treatment CRT at baseline (and follow up) was also associated with insufficient treatment response to other anti-VEGF therapies [107].

Conclusion: CRT thickness positively correlates with VEGF and inflammatory cytokine levels such as IL-6, IL-8, PDGF, and others. A greater CRT seems to be associated with fast ME recurrence under anti-VEGF therapy. The CRT significantly decreased in anti-VEGF non-responders after switching to steroids, while there was no significant effect when switching from steroids to anti-VEGF. These findings suggest that an insufficient decrease in the CRT after anti-VEGF may be linked to a predominantly non-VEGF-driven disease activity, and these patients will probably benefit from a switch to steroids.

**Disorganization of the inner retinal layers (DRIL)** is characterized by the inability to differentiate and segment the boundaries between the ganglion cell layer–inner plexiform layer (GCL-IPL), the inner nuclear layer (INL), and outer plexiform layer (OPL) in the inner central 1 mm. This disruption likely reflects a disruption of the visual pathway between the photoreceptors and ganglion cells. Indeed, the degree of DRIL in the center field is associated with the severity of ischemic damage in RVO, where it correlates with an enlargement of the foveal avascular zone and a disruption of the EZ and ELM [126,136,137,138,139]. DRIL’s association with areas of ischemic damage and loss of flow in the superficial, middle, and deep capillary plexuses, as observed through OCT–angiography (OCT-A), highlights its potential as a biomarker for ischemic injury in RVO [136,140]. The presence and extent of DRIL have been linked to worse baseline BCVA in ME in several macular diseases [119,141,142] and to poorer visual outcomes in RVO [119,120,121]. For instance, in a longitudinal study in eyes with RVO-associated ME treated with anti-VEGF, the absence of DRIL at baseline was associated with greater BCVA gains, and the presence and persistence of DRIL was associated with significantly less BCVA gains at 6 months and 12 months of continuous anti-VEGF treatment [121]. These observations underscore the prognostic significance of DRIL.

Notably, the extent of DRIL at later stages of treatment appears to have greater prognostic value than at baseline. This is evidenced by studies showing that the extent of DRIL at baseline was not linked to the initial visual acuity (VA) in eyes with acute CRVO that were receiving treatment for the first time. However, after a six-month follow-up period, the extent of DRIL at that time point was correlated with poorer VA and proved to be a predictor of worse VA throughout an observation period exceeding two years. Additionally, baseline ischemic features identified through ultra-widefield fluorescein angiography may be indicative of the future development of DRIL, further supporting its role as a marker of ischemic damage [131].

While there are no studies so far that have assessed the course of DRIL as a potential biomarker in RVO-associated ME, a study in DME showed that DRIL was a significant predictor for poor treatment response, representing a significant risk for poor visual recovery to anti-VEGF therapy (odds ratio = 7.05; *p* = 0.034 for DRIL) [143]. Intravitreal steroids are also known to improve DRIL alongside a decrease in HRF and CRT [144].

Conclusion: DRIL is an important prognostic biomarker. Most studies have linked the presence of DRIL to poorer visual outcomes in RVO-associated ME. The absence of DRIL at baseline is associated with better BCVA gains after anti-VEGF treatment, whereas persistent DRIL predicts worse visual acuity over time. There are no studies on the predictive ability of DRIL as a biomarker for treatment response to anti-VEGF in RVO-associated ME. However, in DME, DRIL appears to predict a poor response to anti-VEGF therapy, and steroids might be beneficial in these cases.

**Prominent middle limiting membrane (p-MLM)** appears to be an indicator of ischemic damage and is described as a hyperreflective line at the inner synaptic portion of the outer plexiform layer [145]. Differences in the severity, progression, and duration of retinal hypoperfusion contribute to the development of a multifaceted condition known as the retinal ischemic cascade with a complex spectrum, where the cumulative impact on the middle and inner retina can vary significantly [146]. Paracentral acute middle maculopathy (PAMM) is characterized by hyperreflective band-like lesions, indicating inner nuclear layer (INL) infarction. The INL is particularly susceptible to even minor levels of retinal hypoperfusion and ischemia [146]. PAMM is believed to result from microvascular ischemia within the intermediate capillary plexus. This plexus represents a watershed zone due to its distal location along the retinal vascular pathway [147]. Over time, unnoticed PAMM lesions can become atrophic, leading to areas of INL thinning known as “resolved” PAMM. These resolved lesions may serve as early indicators of an increased RVO and cardiovascular risk profile [148,149].

The p-MLM sign occurs in 28% of cases of ischemic CRVO [150]. This sign has been discussed as a potential prognostic indicator for later anterior segment neovascularization in CRVO patients, but current data do not support its reliable prediction [150]. But, the p-MLM sign is associated with poorer visual outcomes and a higher likelihood of an ischemic CRVO [122]. Another study identified p-MLM signs and microaneurysms as risk factors for the failure to maintain extended anti-VEGF treatment intervals [108].

An increased formation of microaneurysms and the presence of p-MLM may indicate more severe ischemic damage, resulting in elevated VEGF concentrations. This suggests that anti-VEGF treatment might be appropriate, though it may require high frequencies, as the VEGF pathway appears to be the primary component. Currently, there are no studies that have investigated the association between p-MLM and inflammatory cytokines in ME of any origin. Further research is necessary to explore the effects of steroids in this context.

Conclusion: The p-MLM sign indicates ischemic damage with significantly elevated VEGF levels. Data suggest that this sign is a risk factor for the failure to maintain extended anti-VEGF treatment intervals. Patients with the p-MLM sign might respond well to anti-VEGF therapy but may require higher doses and more frequent treatments. More data are needed to confirm these hypotheses.

**The ellipsoid zone:** The integrity of the ellipsoid zone (EZ) provides a unique opportunity to assess the overall integrity and “health status” of the photoreceptor outer segments [151]. The outer retina is characterized by four highly reflective bands visible on spectral domain OCT: the external limiting membrane (ELM), ellipsoid zone (EZ), interdigitation zone (IZ), and retinal pigment epithelium (RPE). The integrity of the EZ is strongly associated with the maintenance of visual acuity in eyes with RVO and macular edema [123]. An intact EZ is correlated with better BCVA during a 12-month follow-up [124]. Treatment with anti-VEGF leads to an improvement in the integrity of the EZ [152]. Ellipsoid zone disruption (EZD) is correlated to the degree of ischemia and response to treatment [153,154]. An intact EZ was associated with favorable treatment response after 12-month anti-VEGF therapy in patients with treatment-naïve ME [109], while a loss of integrity at the EZ junction was more frequent in poor responders to anti-VEGF treatment [110]. In such cases, a dexamethasone implant may promote the resolution of macular edema but it likely is not associated with an improvement in visual function [5,111]. Loss of EZ integrity, especially when it is present after ME resolution, is a poor prognostic factor. However, with the long-term use of intravitreal steroids despite EZ disruption and nonperfusion of the outer retina, some recovery has been reported. In a study in which repeated injections were given for 5 years, dexamethasone-treated eyes showed better retinal perfusion, a higher FAZ circularity index, and a higher retinal perfusion density, especially in the deep capillary plexus, than anti-VEGF-treated eyes [5].

Conclusion: Loss of central EZ integrity is a poor prognostic sign and is a likely indicator of poor visual acuity gain under therapy. It may also suggest an inferior anti-VEGF response. Patients with disrupted EZ in RVO-associated ME may preferentially benefit from intravitreal steroids. The VA benefits may be limited or significantly delayed despite resolution of fluid.

**Location of intraretinal cysts**: Tilgner et al. investigated the characteristics of intramacular cysts appearing in patients with a CRT of <250 micrometers following 1–4 anti-VEGF injections and a dry macula. In 58.5% of these eyes, at the next visit, 4 to 6 weeks later, macular intraretinal cysts were observed even in the presence of a CRT of <250 micrometers. During follow up, these patients exhibited an enlargement of cystic changes, leading to an increase in CRT, poor BCVA, and a recurrence of an ME > 250 µm [112]. Cysts in the ganglion cell layer had the highest negative prognostic value for visual acuity, indicating permanent damage to the inner retina due to RVO, and ganglion cell loss [125]. Thus, persistent intraretinal cysts, despite a satisfactory central retinal thinning, is a predictor of either ongoing disease activity or permanent structural degeneration. Although there is no evidence that steroids prevent this recurrence, steroid implants are longer-acting and may have a beneficial effect in preventing disease activity, significant retinal thickening, and BCVA decrease. The impact of GCL cysts as a poor prognostic and predictive biomarker in retinal vascular diseases treated with anti-VEGF have been seen in another study, where patients with DME were treated with anti-VEGF [155]. In the presence of GCL cysts, BCVA was significantly decreased, and eyes also showed less treatment response in terms of the CRT decrease. Another study assessed the mean retinal sensitivity via microperimetry in relation to the mean cyst area and found that the GCL cyst area had by far the most severe impact on the mean retinal sensitivity decrease (in mean by -6 dB) [156].

Conclusion: The presence of intraretinal cysts, especially within the ganglion cell layer (GCL), serves as a negative prognostic indicator for visual acuity recovery following anti-VEGF treatments. These cysts are associated with worsening CRT, poor BCVA, and the recurrence of ME, signifying ongoing disease activity despite initial treatment success. In particular, GCL cysts may indicate inner retinal damage, chronicity, and ischemia, leading to decreased BCVA and reduced response to treatment in terms of CRT reduction. Comparative studies are needed, but intravitreal steroids could offer benefits in these chronic cases with persistent intraretinal cysts.

**Hyperreflective foci (HRF)** are defined as small, discrete, well-circumscribed, dot-shaped lesions with equal-or-greater reflectivity than the RPE on SD-OCT. They need to be differentiated from hard exudates (HEs). HRF are believed to represent activated microglial cells. The size varies, but usually it ranges from a few micrometers to twenty micrometers. HEs, in contrast, appear as highly reflective lesions, often with shadowing that obscures the retina below, and they are larger than HRF [157]. HRF can be located in any retinal layer [158]. In retinal vascular diseases, HRF have been shown to have a strong prognostic and predictive value for treatment response. They are an indicator of active inflammation, as they correlate especially with inflammatory cytokines such as CD14, IL-1beta, and IL-6 which stimulate microglial cells. The presence of HRF in RVO-associated ME is associated with an increasing CRT and IRF, the presence of SRF, and the disruption of the EZ [159]. In inflammatory states, microglia tend to accumulate along the RPE. The accumulation of HRF in the outer retina has been observed in RVO and was associated with a poor visual outcome [158].

In DME, a large number of HRF (usually defined as >20 HRF) has been shown to be a general poor prognostic marker for treatment response with anti-VEGF [113,119]. In RVO, the absence of HRF at baseline was associated with a favorable treatment response to anti-VEGF therapy after 12 months [109]. A significant decrease in the number of HRF in persistent ME following anti-VEGF treatments can be observed with a positive correlation between IL-8 levels and the number of HRF observed on SD-OCT [68]. When comparing the effect on HRF in RVO with anti-VEGF or steroids (Ozurdex), the latter appears to be more effective in reducing the number of HRF [114]. With both agents, such a reduction is correlated with an improvement in vision [159]. In another study of 139 patients, a greater reduction in the number of outer retinal HRF and better BCVA were achieved with a dexamethasone implant after 3 months of therapy compared to the treatment group with anti-VEGF [115]. Some authors have suggested that in the presence of higher HRF numbers, intravitreal steroids should be considered [116,117].

Conclusion: The presence of numerous HRF is indicative of an elevated inflammatory state in retinal vascular diseases. Generally, a high number of HRF is considered a poor prognostic indicator in macular edema associated with RVO. However, these foci may be predictive of a favorable response to intravitreal steroids compared to anti-VEGF therapies.

**Subretinal fluid (SRF)** serves as a prognostic biomarker in RVO for BCVA. SRF is associated with higher concentrations of inflammatory cytokines such as IL6, MCP-1, and ICAM-1 [160]. The inflammatory component may be the driving force to induce a serous retinal detachment, and SRF secondary to CRVO suggests a primary inflammatory component [161,162,163,164,165,166,167]. The significantly higher prevalence of SRF in primarily inflammatory-driven retinal pathologies, such as uveitic cystoid macular edema (CME) and Irvine–Gass syndrome, compared to its lower prevalence in RVO-associated CME, suggests that SRF is predominantly driven by a higher inflammatory state in the eye [168]. The resolution of the different fluid compartments (sub- or intraretinal) also appears to vary based on the level of inflammation. In DME, SRF resolves first after treatment, whereas in uveitic CME, SRF resolution is much slower, and intraretinal fluid (IRF) typically resolves before SRF [168,169]. SRF can be found in up to 70% of acute RVO-associated ME [124]. The rate of resolution of this SRF occurs faster in patients receiving anti-inflammatory therapies (Ozurdex) as compared to anti-VEGF (aflibercept, ranibizumab) [114]. The sole presence of SRF is not yet a prognostic factor and a predictive factor for inferior treatment response to anti-VEGF. While the initial presence of SRF is not yet an informative biomarker in treatment-naïve RVO-associated ME, the initial response to treatment may provide important insights [119,170,171]. The persistence of SRF despite treatment indicates a more chronic form of the disease and also higher levels of inflammatory cytokines [124]. In these cases, intravitreal steroids may have a better effect on fluid resolution than anti-VEGF treatment. However, more studies are needed here to understand the role of SRF as predictive biomarker in RVO-associated ME.

Conclusion: The presence of SRF is very prevalent in acute RVO-associated ME. While the sole presence is not a poor prognostic or predictive sign, the persistence of SRF may indicate chronicity and a higher inflammatory state, and intravitreal steroids may have a beneficial effect in these cases.

**Choroidal thickness:** The choroid plays a crucial role in regulating the metabolism of the RPE and the outer retina. An increased **choroidal thickness** (>300 μm), often accompanied by dilated choroidal vessels and other structural changes, is a predictive OCT biomarker for a poor response to anti-VEGF therapy [118]. The reason for this is not clear, but a thickened choroid may be a sign for the predominant presence of proinflammatory cytokines, such as IL-4, IL-6, IL-8, MCP-1, and IP-10, which are not affected by anti-VEGF. Interestingly, in central serous chorioretinopathy and pachychoroid neovasculopathy, some of these cytokines are also upregulated [172,173].

Conclusion: A subfoveal choroidal thickness of >300 micrometers prior to treatment start may be a sign of a less favorable treatment response to anti-VEGF. As inflammatory cytokines are associated with an increase in choroidal thickness, intravitreal steroids may be beneficial.

#### 3.4.2. Features in Optical Coherence Tomography (OCT)–Angiography (OCT-A)

OCT-A is a simple and noninvasive method for imaging retinal capillaries without the injection of contrast dyes. It allows for visualization and differentiation between both the superficial capillary plexus (SCP) and deep capillary plexus (DCP) and reveals impaired vascular perfusion in both plexus, enlargement, and acircularity of the FAZ and microvascular abnormalities.

It should be noted that the presence of ME significantly reduces the validity and reproducibility of OCT-A measurements. Particularly, the reliability of OCT-A appears to be poor when the central macular thickness exceeds 400 μm [174]. ME can cause segmentation errors due to the distortion of retinal layers, which negatively impacts vascular density (VD) measurements. Additionally, lower VA in these patients can lead to fixation issues, increasing motion artifacts. Furthermore, the edema can obscure the retinal vasculature, and this overshadowing effect, combined with other artifacts, makes it difficult to obtain high-quality scans, complicating the comparative analysis of OCT-A biomarkers. Many previous OCT-A studies on RVO patients have included eyes with ME without adequately considering its impact on VD measurements, raising concerns about the reliability of their results.

**Vessel density (VD) and Fractal Dimension (FD)** are OCT-A markers that quantify retinal vasculature. VD is the most widely available quantitative biomarker in OCT-A. It reflects the percentage of the sample area occupied by vessel lumens [175,176]. FD measures the vascular complexity and branching of the retinal vasculature. Following RVO, low values of FD and a decrease in VD are observed in both the superficial and deep plexus, with a more pronounced impact on the DCP [175,176,177,178]. The DCP is highly dependent on perfusion pressure. It is primarily formed by venous collecting channels and may be more susceptible to vascular occlusion, higher interstitial pressure and subsequent ischemia. It has less collateral blood flow support compared to the SCP. The DCP supplies the watershed zone located between the inner nuclear layer (INL) and the outer plexiform layer (OPL), including neuronal synapses that transfer vision signals from photoreceptors to ganglion cells [177]. The preservation of the DCP appears to correlate with better VA outcomes when it is shown to be intact initially [179]. This association holds true for both BRVO [159] and CRVO [180,181]. Low VD and low FD in the DCP were found to be significantly associated with poor visual acuity in RVO. It is obvious that OCT-A parameters such as FD and VD, which theoretically indicate the extent of macular nonperfusion, would be linked to visual function. Reduced VD can arise from either diminished perfusion flow or capillary rarefaction, whereas decreased FD is attributed to capillary dropout. In eyes affected by RVO, capillary dropout may coincide with compensatory vessel dilatation, resulting in a more significant decrease in FD compared to VD. This observation suggests that FD in the DCP may serve as the more reliable prognostic factor for BCVA in RVO-affected eyes [178].

Eyes in which VD is maintained experience fewer recurrences of ME due to BRVO [182,183]. Tomita et al. studied 29 eyes with ME due to BRVO under anti-VEGF therapy and found that the mean VD reduction in eyes with ME resolution after one anti-VEGF injection was significantly higher than in eyes with recurrent ME (*p* = 0.028) [184]. The reason is not clear; one suggestion by the authors was that a great reduction in macular vasculature may eliminate the source of leakage, leading to fewer ME recurrences [185]. However, eyes with significant macular capillary defects may suffer from inner retinal layer atrophy, resulting in lower oxygen demand and VEGF production [182,184,186,187]. This observation will definitely warrant further studies.

Conclusion: Low VD and low FD are associated with poor visual acuity in RVO at the initial diagnosis and appear to have prognostic value. A reduction in VD may predict a good response to anti-VEGF therapy in terms of fluid resorption, but it may also indicate atrophy of the inner retinal layer, resulting in lower oxygen demand and reduced anti-VEGF production, leading to worse functional outcomes. Data suggest that VD could be a valuable parameter for predicting the number of anti-VEGF injections needed for ME secondary to BRVO, as it shows a significant negative correlation with the total number of intravitreal injections required. A lack of reduction in VD after one anti-VEGF injection may indicate the need for a high frequency of anti-VEGF treatments. Due to chronicity, these cases may also benefit from intravitreal steroids.

**Nonperfusion Areas (NPAs),** microaneurysms, and microvascular changes in RVO are more frequently seen in the DCP than in the SCP [175,188,189]. The presence of NPAs in OCT-A correlates with peripheral nonperfusion [175] and therefore has prognostic value. OCT-A findings in BRVO confirmed that central foveal and parafoveal nonperfusion, especially in DCP, correlated with more recurrent ME after intravitreal bevacizumab therapy [190]. Suzuki et al. showed that after 6 months of anti-VEGF therapy (ranibizumab and aflibercept) in RVO-ME, there was a reduction in NPAs and an improvement in retinal blood flow, especially in the deep plexus [191]. After successful anti-VEGF therapy, visual rehabilitation was associated with a better perfusion of the SCP and DCP [181]. However, there are also reports in which no improvement in macular perfusion were seen with therapy [140]. One study, which was already cited in the VD section, showed that in cases of BRVO, eyes with more restricted macular perfusion experience fewer recurrences of edema and require fewer intravitreal anti-VEGF injections. Preserved ischemic capillaries may produce more VEGF than non-perfused capillaries, also known as ghost capillaries [182]. So far, head-to-head studies with intravitreal steroids are lacking. In 33 patients with treatment-naïve DME, flow voids did not significantly change under either anti-VEGF (ranibizumab) or steroid (dexamethasone) treatment. Significant changes in the perfusion density (PD) in the DCP were found; two months after receiving intravitreal dexamethasone treatment, the PD decreased, while subsequent to three monthly injections of ranibizumab, the following month, PD was noted to be higher [192]. Numerous inflammatory cytokines (IL-6, IL-8, IL-12, IL-13, MCP-1, ICAM-1) are elevated in RVO patients with NPAs, yet the predictive ability of NPAs concerning treatment decisions between anti-VEGF and steroids in RVO-related ME remains unclear. [26,57,69,81].

Conclusion: In patients with NPA, there is a relevant hypoxic pathophysiologic pathway with significant elevated VEGF levels. Elevated levels of inflammatory cytokines are also observed. Therefore, both the anti-VEGF and anti-inflammatory approaches are sound and justified. Certain studies support the use of anti-VEGF to reduce the size of NPAs and improve retinal blood flow in the SCP and DCP. Data from treatment-naïve DME patients suggest improved perfusion in the DCP following anti-VEGF treatment compared to steroids one month after injections, but this has yet to be demonstrated in RVO. Without head-to-head studies in patients with RVO-related ME, NPAs cannot yet be used as a predictive biomarker in these cases.

**The foveal avascular zone (FAZ)** is enlarged in both ischemic CRVO and BRVO [193]. The FAZ size seems to be negatively correlated with BCVA in patients with RVO-associated ME [139,194]. A higher “acircularity index” [195], representing a measure of the irregularity at the boundary of the FAZ, also provides a negative index. After successful anti-VEGF therapy, there was a clear association between visual rehabilitation and a reduction in the size of FAZ in both plexuses [181]. Suzuki et al. observed changes in the FAZ after anti-VEGF treatment in both the SCP and DCP. Notably, eyes that received fewer anti-VEGF injections exhibited a greater degree of FAZ enlargement. In eyes with more frequent injections, the FAZ size in both the layers was much smaller compared to eyes with fewer injections [191]. However, other data show that the FAZ area remained statistically unchanged before and after anti-VEGF therapy (bevacizumab, ranibizumab, aflibercept) [196]. Interestingly, data of patients with treatment-naïve DME showed a greater decrease in the FAZ circularity index in those who received intravitreal dexamethasone compared to those who received three monthly injections of ranibizumab. The FAZ circularity index was retrospectively evaluated at baseline and compared 2 months after intravitreal dexamethasone injection (15 eyes) and 1 month after three monthly ranibizumab injections (18 eyes) [192].

Conclusion: There are substantial data on the FAZ and perfusion changes after RVO and subsequent anti-VEGF therapy. However, research on the relationship between the FAZ area and ME recurrence is limited. Enlargement of the FAZ might be related to intraocular VEGF levels and, therefore, could potentially predict the response to anti-VEGF treatment. Further studies are needed to explore this relationship.

#### 3.4.3. Features on Fluorescein Angiography (FA)

OCT-A lacks the capability to evaluate changes in vascular permeability or leakage. Therefore, FA plays an important role to assess vascular leakage in RVO patients, which is crucial for assessing macular edema and neovascularization. In addition, it provides a detailed view of the retinal perfusion status, allowing for the assessment of both macular and peripheral nonperfusion. This information is vital for understanding the extent of ischemia. FA captures the dynamic process of blood flow through the retinal vasculature, offering insights into the timing and pattern of leakage and perfusion abnormalities.

**FA leakage** indicates loss of tight junctions and impaired functionality of blood vessels [197]. In BRVO, increased FA leakage correlates with increased post-treatment CRT, CRT at one year, and a higher number of anti-VEGF injections within the first year of treatment [107]. Severe fluorescein leakage suggests more serious damage to the integrity of retinal vessels and is associated with refractory or recurrent macular edema. Fluorescein leakage observed on FA results from increased vascular permeability. Besides VEGF over-expression, various other pathways, such as elevated inflammatory cytokines (IL-6, IL-8, TNF-alpha, ICAM-1, MCP-1, PDGF, TGF-beta, MMPs, Ang-2, ET-1, etc.), leukostasis, alterations in intercellular junctions, and impairment of the neurovascular unit can also increase vascular permeability [24,47,73,75,198]. For these pathways, corticosteroids might offer a beneficial treatment alternative.

The study also highlighted the significance of the location of the affected vessels. For instance, extramacular BRVO cases with large areas of non-perfusion and FA leakage outside the macula may sometimes lead to neovascular complications but rarely cause macular edema or respond well to anti-VEGF. In contrast, BRVO cases that affect only a small portion but with localized FA leakage within the macula tend to result in refractory macular edema [107].

Conclusion: Extensive fluorescein leakage, particularly within the macula, is linked to an inferior response to anti-VEGF treatment and persistent ME after BRVO. This may be due to additional inflammatory pathways involved. The chronic nature and inflammation in these cases suggest a potential benefit of a switch to steroids, although further studies are needed to confirm their efficacy.

**Macular ischemia** is recognized as a negative prognostic factor for visual outcome [127,128,129]. Ischemic eyes with RVO have worse functional outcomes in long-term follow up [127,128]. In a study by Chatziralli et al., intravitreal anti-VEGF treatment was effective in ischemic CRVO, stabilizing non perfusion and enhancing VA. However, it did not necessarily lead to visual improvement in cases with macular ischemia over the 24-month follow-up period [129]. Another study found that the absence of macular ischemia was associated with a favorable response to anti-VEGF therapy after 12 months in treatment-naïve ME patients [109] Conversely, the CRYSTAL study, encompassing both ischemic and non-ischemic CRVO patients, indicated that the improvement in VA after treatment with ranibizumab was independent of macular ischemia at baseline [130]. Findings from other anti-VEGF studies also indicate that patients with retinal nonperfusion or macular ischemia continue to benefit from anti-VEGF therapy [18,19,199]. Macular ischemia at baseline appears to have a minimal effect on functional outcomes and the need for re-treatment. Elevated inflammatory cytokine levels (e.g., MCP-1) in RVO-ME patients with macular ischemia highlight the inflammatory component in these cases, which will require further study [81].

Conclusion: The visual prognosis in CRVO eyes with macular ischemia is worse than in non-ischemic eyes. While baseline macular ischemia appears to have only a minimal effect on the need for anti-VEGF re-treatment, severe cases may benefit from steroids. Long-term studies show that intravitreal dexamethasone can improve retinal perfusion compared to anti-VEGF [5], though VA gains in severe ischemia may be limited.

#### 3.4.4. Aqueous Flare

Anterior chamber flare is commonly used in the management of intraocular inflammation and to determine the level of blood ocular barrier integrity. Laser flare photometry uses the scattering of a low-energy laser beam to quantify the amount of protein in the anterior chamber. Anterior chamber flare is increased in RVO and correlates with levels of intraocular inflammatory cytokines [23,200]. Considering that intravitreal steroids successfully suppress respective proinflammatory proteins, a higher anterior chamber flare may suggest a beneficial effect of intravitreal steroids compared to anti-VEGF treatment. However clinical studies would be needed to confirm this assumption.

## 4. Discussion

In this review, we identified biomarkers relevant to RVO in patients with ME. This review explored biomarkers from different imaging modalities and cytokine assessments, delving into their collective predictive potential concerning the therapeutic efficacy of anti-VEGF and steroid treatments.

Intravitreal steroids are superior to anti-VEGF in a subset of RVO-associated ME patients, particularly those with inflammation-driven or chronic ME. Numerous inflammatory cytokines are increased in ME secondary to RVO. Positive correlations were found between the intraocular levels of several cytokines (IL-6, IL-8, MCP-1, ICAM-1, and ANG2) and CRT, NPAs [26,57,69,81], serous retinal detachment [73,170], and ME severity [24,49].

The ability to link these cytokines and pathways with corresponding imaging biomarkers could enhance therapeutic decision-making.

Evaluating morphological biomarkers both at baseline and during the treatment course can assist in formulating the treatment strategy at the initiation of therapy. It may also aid in deciding whether an early transition to steroid treatment is warranted. The prognosis of CRVO deteriorates with the persistence of macular fluid beyond this period [201]. Therefore, a timely transition to a dexamethasone implant in suitable patients is crucial to prevent irreversible retinal cell loss due to persistent edema. Utilizing biomarkers in RVO-ME becomes crucial for distinguishing between high and low treatment demands, aiding in predicting potential responses to diverse treatment modalities.

For the initial assessment of patients with ME secondary to RVO, baseline imaging should include SD-OCT and OCT-A. These imaging techniques are essential for evaluating the presence of HRF, SRF, CRT, choroidal thickness, and the integrity of the FAZ. Additionally, FA should be conducted to assess the degree of vascular leakage and the macular perfusion status.

Anti-VEGF therapy is typically indicated for patients without significant inflammatory biomarkers, such as HRF and persistent SRF, as they are likely to respond well to this treatment [68,109,114,124,160]. Patients with minimal FA leakage in the macula, as well as those showing significant CRT reduction and VA improvement after initial anti-VEGF injections, are good candidates for this therapy [59,105]. Regular monitoring with OCT is recommended to assess the treatment response, and the frequency of injections should be adjusted based on CRT reduction and VA improvement, with the possibility of extending the intervals if stability is achieved.

Steroid therapy is indicated for patients with elevated inflammatory biomarkers, the presence of HRF, and persistent SRF detected by SD-OCT, indicating elevated inflammation or chronicity. A choroidal thickness greater than 300 μm suggests underlying inflammation and a poor response to anti-VEGF therapy [118]. Patients who show a minimal decrease in CRT after anti-VEGF therapy should be considered for an early switch to steroids [59,105]. Additionally, severe FA leakage in the macula indicates poor response to anti-VEGF, possibly due to additional inflammatory pathways [107]. An early reassessment post dexamethasone implant is necessary to monitor changes in CRT and VA, and the implant may be repeated as needed, typically around six months, based on clinical response.

Prognostic indicators play a crucial role in guiding treatment decisions. Poor prognostic signs, such as intraretinal cysts within the ganglion cell layer, disintegrity of the central EZ, and DRIL are associated with limited VA recovery [110,111,119,120,121,125,154]. In these chronic cases, steroids may offer benefits, though VA improvement may be limited. OCT-A can be utilized to assess the FAZ integrity and VD. Data suggest that VD could be a valuable parameter for predicting the number of anti-VEGF injections needed for ME secondary to BRVO. One thesis here is that a severe reduction may eliminate the source of leakage, but patients may also suffer from capillary defects and inner retinal atrophy with limited visual prognosis [184].

Long-term management should consider the potential for retinal perfusion improvements with dexamethasone implants compared to anti-VEGF therapy [5]. However, VA gains in cases of severe ischemia may be limited. It is essential to consider the recurrence of ME and the role of inflammation in its pathophysiology when adjusting treatment plans.

Research on the relationship between the FAZ area, NPAs, and ME recurrence is limited. In-depth insights into vascular morphology and the effects of non-perfusion and ischemia on ME via advanced OCT-A diagnostics are needed. These insights can further aid in understanding the pathophysiology of macular edema in RVO, particularly in conjunction with cytokine interactions. OCT-A diagnostics show promising potential, especially with advancements in machine learning. However, the presence of concurrent ME significantly affects the validity and reproducibility of these measurements, emphasizing the need for ongoing research to refine these biomarkers for clinical use. Further head-to-head studies with steroids and anti-VEGF using OCT-A technology are necessary to identify reliable predictive OCT-A biomarkers.

In the coming years, advancements in machine learning techniques are poised to enhance personalized treatment strategies by leveraging a vast array of biomarkers. These approaches will involve weighing the relevance of individual features and their interactions to identify the most effective treatment choices. Foundation models will help to integrate different types of data and will allow for training from text and imaging data. Patients with RVO who exhibit biomarkers indicating a generally unfavorable response to existing treatments may potentially derive benefits from innovative drugs targeting additional pathways within the intricate pathophysiology of RVO.

Emphasis should be placed on the investigation of morphologic and inflammatory factors that induce ME in RVO cases. SD-OCT and OCT-A should aid in identifying specific morphological biomarkers, guiding the choice between first-line intravitreal medications (anti-VEGF versus corticosteroids). Additionally, it is imperative to investigate the interplay of cytokines that activate both pertinent risk factors and RVO itself. These cytokines play pivotal roles in elucidating the disease mechanism and could emerge as promising targets for treatment. Circulating biomarkers as well as retinal imaging markers have the potential to facilitate more personalized treatment approaches, leading to improved visual outcomes. Artificial intelligence and platforms such as Alphafold may also help here and could be used to explore the potential pathways.

## Figures and Tables

**Table 2:** Predictive biomarkers regarding treatment response to anti-VEGF or steroids in patients with ME due to RVO.

Predictive Biomarkers	Predictor of Good Response to Anti-VEGF	Predictor of Good Response to Steroids	References
Thicker (>570 µm) CRT at baseline and/or at follow up	NO	YES	[104,105,106,107]
p-MLM sign	no data, may indicate high treatment need for anti-VEGF	no data	[108]
Loss of EZ integrity	NO	no data	[5,109,110,111]
Presence of intraretinal cysts	NO	no data	[112]
HRF	NO	YES	[109,113,114,115,116,117]
Persistent SRF	NO	YES	[113]
Choroidal thickness >300 micrometers	NO	no data	[118]
Extensive FA leakage within the macula	NO	no data	[107]
Higher levels of VEGF and EPO	YES	NO	[24,52,59,64,97,99]
Higher aqueous levels of IL-6, IL-12, IL-13, MCP-1, ICAM-1, IP-10, PTX 3	NO	YES	[27,30,49,52,57,58,59,65,69,70,71]

Abbreviations: CRT: central retinal thickness; p-MLM: prominent middle limiting membrane; EZ: ellipsoid zone; HRF: hyperreflective foci; SRF: subretinal fluid; FA: fluoresceine angiography; VEGF: vascular endothelial growth factor; EPO: erythropoietin; IL: interleukin; MCP: monocyte chemoattractant protein; ICAM: intercellular adhesion molecule; IP: interferon protein; PTX: pentraxin; ME: macular edema; RVO: retinal vein occlusion.

**Table 3:** Prognostic biomarkers for poor visual outcome in patients with ME due to RVO.

Prognostic Biomarkers for Poor Visual Outcome	References
Greater extent of DRIL (greater later in the course of treatment)	[119,120,121]
Presence of p-MLM sign	[122]
Loss of EZ	[123,124]
Presence of intraretinal cysts (especially in the GCL)	[112,125]
Presence of HRF	[126]
Macular non-perfusion on FA	[127,128,129]

Abbreviations: DRIL: disorganization of the inner retinal layers; p-MLM: prominent middle limiting membrane; EZ: ellipsoid zone; GCL: ganglion cell layer; HRF: hyperreflective foci; FA: fluorescein angiography; ME: macular edema; RVO: retinal vein occlusion.

## Data Availability

No new data were created.
